# Dreyfus scale-based feedback increased medical students’ satisfaction with the complex cluster part of a interviewing and physical examination course and improved skills readiness in Taiwan

**DOI:** 10.3352/jeehp.2019.16.30

**Published:** 2019-10-11

**Authors:** Shiau-Shian Huang, Chia-Chang Huang, Ying-Ying Yang, Shuu-Jiun Wang, Boaz Shulruf, Chen-Huan Chen

**Affiliations:** 1 Department of Medicine, National Yang-Ming University, Taipei, Taiwan; 2 Bali Psychiatric Center, Ministry of Health and Welfare, Taipei, Taiwan; 3 Division of Clinical Skills Training, Department of Medical Education, Taipei Veterans General Hospital, Taipei, Taiwan; 4 Division of General Medicine, Department of Medicine, Taipei Veterans General Hospital, Taipei, Taiwan; 5 Office of Medical Education, University of New South Wales Australia, Sydney, Australia; Hallym University, Korea

**Keywords:** Cohort studies, Group structure, Medical students, Personal satisfaction, Physical examination, Taiwan

## Abstract

**Purpose:**

In contrast to the core part of the clinical interviewing and physical examination (PE) skills course, corresponding to the basic, head-to-toe, and thoracic systems, learners need structured feedback in the *cluster* part of the course, which includes the gastrointestinal, neuromuscular, and musculoskeletal systems. This study evaluated the effects of using Dreyfus scale-based feedback, which has elements of continuous professional development, instead of Likert scale-based feedback in the cluster part of training in Taiwan.

**Methods:**

Instructors and final-year medical students in the 2015–2016 classes of National Yang-Ming University, Taiwan comprised the regular cohort, whereas those in the 2017–2018 classes formed the intervention cohort. In the intervention cohort, Dreyfus scale-based feedback, rather than Likert scale-based feedback, was used in the cluster part of the course.

**Results:**

In the cluster part of the course in the regular cohort, pre-trained standardized patients rated the class climate as poor, and students expressed low satisfaction with the instructors and course and low self-assessed readiness. In comparison with the regular cohort, improved end-of-course group objective structured clinical examination scores after the cluster part were noted in the intervention cohort. In other words, the implementation of Dreyfus scale-based feedback in the intervention cohort for the cluster part improved the deficit in this section of the course.

**Conclusion:**

The implementation of Dreyfus scale-based feedback helped instructors to create a good class climate in the cluster part of the clinical interviewing and PE skills course. Simultaneously, this new intervention achieved the goal of promoting medical students’ readiness for interviewing, PE, and self-directed learning.

## Introduction

### Background

Clinical interviewing and physical examination (PE) skills are the beginning of the patient-doctor relationship, and comprise diagnostic steps, diagnosis, and treatment choices. The development of strong interviewing and PE skills among health professionals improves the quality of care and decreases healthcare costs [1]. The recent literature has revealed declining interviewing and PE skills among graduates [1,2]. Year-long small group-based sessions for interviewing and PE skills for final-year medical students regularly begin with a simple core part and conclude with a complex *cluster* part [2]. The performance of medical students in the complex cluster part of interviewing and PE training has been reported to be poorer than their performance in the basic core part. In fact, with the increasing complexity of the cluster part, a scaling format with elements of progression in expertise is crucial for enhancing the effectiveness of instructor feedback. Furthermore, work-time restrictions and a scarcity of time for bedside clinical teaching make structured feedback necessary to increase the efficiency of skill training [3].

In recent years, medical education has emphasized the role of self-directed learning (SDL) in establishing clinical competencies after receiving structured feedback [3]. For medical students, success in interviewing and PE training is dependent on the quality of feedback from instructors [4]. Structural descriptions of competency levels of interviewing and PE skills are needed for instructors and students [5]. The Dreyfus 5-stage model clearly divides competency levels into novice, advanced beginner, competent, proficient, and expert to present the progression of expertise in clinical and SDL skills [6]. Using the Dreyfus scale, advanced medical students are expected to be at the advanced beginner level for clinical skills [7].

### Purpose

This study aimed to explore the effects of implementing Dreyfus scale-based feedback by instructors on the satisfaction, performance, and SDL skills of final-year medical students at National Yang-Ming University, Taiwan. Specifically, it evaluated the effects of using the Dreyfus scale, which has elements of continuous professional development, instead of a Likert scale for feedback in the cluster part of training.

## Methods

### Ethics statement

Ethical approval was obtained from the ethics committee of our institution (IRB approval no., 2015-06-001B) and care was taken to apply the principles of the World Medical Association Declaration of Helsinki to the research. Oral informed consent was obtained from subjects.

### Study design

This was a cohort study with a comparative analysis of groups based on survey results from March 2015 to March 2019 (Dataset 1).

### Setting/participants

#### Content of the interviewing and PE course in the regular cohort

The interviewing and PE skills course began immediately after the summer vacation following the end-of-third-year final written exam. The modified 3 core sessions of the course included basic aspects such as taking vitals, the head, eyes, ears, nose, and throat (HEENT) examination, and the head-to-toe and thoracic systems, whereas the modified cluster sessions focused on the gastrointestinal, neuromuscular, and musculoskeletal systems [2]. Overall, the course included the corresponding lectures and clinical reasoning sessions for cardiovascular and thoracic medicine, as well as the gastrointestinal, neurological, and musculoskeletal systems. At 2- to 3-week intervals, in each hands-on session of the interviewing and PE course, small groups of learners took turns practicing the corresponding skills repeatedly. This was followed by a class demonstration.

Checklists and videos of interviewing and PE skills for specific topics (e.g., the HEENT examination, the head-to-toe system, thorax, abdomen, etc.) were distributed to all students and instructors at least 2 days prior to the learning session, and were also provided during the session. Students were randomly divided into small groups with 9 peers and 2 instructors. The instructors introduced a clinical scenario and then demonstrated the interviewing and PE skills on standardized patients (SPs). In small groups, students took turns practicing their interviewing and PE skills, and feedback was provided by instructors after a checklist-based evaluation of a learner’s performance. In the class demonstrations, instructors were responsible for giving face-to-face feedback to learners after an evaluation using a Likert scale in the regular cohort and the Dreyfus scale in the intervention cohort. The baseline group objective structured clinical examination (GOSCE) was administered after the core part of the course, whereas the end-of-course GOSCE was administered after the cluster part.

#### Background for the integration of Dreyfus scale-based feedback in the complex cluster part of the course

Among the instructors and final-year medical students in the 2015–2016 class, the learner-instructor interactions and student satisfaction with the course and instructors were good for the basic core part. However, less student-instructor interaction and low student satisfaction with the course and instructors were reported in the cluster part. A detailed analysis of the descriptive feedback from medical students revealed that the Likert scale-based feedback did not fully build their readiness for interviewing, PE, and SDL. In the intervention cohort, Dreyfus scale-based feedback was implemented in the cluster part. This intervention helped instructors increase the level of clinical skills development, increase engagement, and improve the classroom climate.

#### Grouping

The instructors and 162 final-year medical students (18 groups in total, with 9 students in each group) in the 2015–2016 classes (9 groups in each year) were enrolled as the regular cohort. The intervention cohort comprised 162 students from the 2017–2018 classes (9 groups in each year, 18 groups in total). There were no significant differences between the students in the 2 cohorts in terms of their average scores on their end-of-third-year final written exam and baseline GOSCE score. Differences between the regular and intervention cohorts in medical students’ performance on the end-of-course GOSCE, learners’ satisfaction with the instructors and course, and students’ readiness for interviewing, PE, and SDL were compared. The instructors’ demographic information is presented in Table 1.

#### Survey of students’ satisfaction with the instructors and course

At the end of the core and cluster parts of the course, students reported their satisfaction with the quality of the instructors’ teaching and the course. Students’ degree of satisfaction with their instructors was measured as the average of the following 6 items: enhancing medical knowledge, teaching materials (such as multimedia), expression and communication, engaging students in learning, expressing care and respect, and quality. Their degree of course satisfaction was measured as the average of the following 6 items: meeting the course objectives, organization, schedule, appropriateness of difficulty, time management, and learning values.

#### The Likert-scale feedback form used among the regular cohort

As shown in Table 2, the Likert-scale form was used for the in-class, baseline, and end-of-course GOSCE evaluations and feedback in the regular cohort. The 6 items related to students’ performance ratings on the Likert scale were scored on a range from 1=poor to 5=excellent.

#### The Dreyfus-scale feedback form used in the intervention cohort

Whereas the Likert scale-based feedback form was used in the core part for both cohorts, the Dreyfus scale-based feedback form was used only in the cluster part for the intervention cohort. In the Dreyfus scale-based feedback, interviewing and PE skills were divided into “doing a comprehensive interview,” including communication/care skills, interviewing techniques, appropriateness of the interviewing sequence, time management for interviewing, and symptom- and laboratory data-based interviewing, and “performing a full PE,” which included examination/care skills, PE techniques, appropriateness of the PE sequence, time management for PE, and symptoms and laboratory data-based PE. Then, the performance of the intervention cohort in the cluster parts was rated using the Dreyfus scale, which corresponded to progression from the novice level to the advanced beginner, competent, proficient, and expert levels for skills acquisition [6,7]. The Dreyfus scale was chosen to reflect the continuum of development along the path of expertise. The performance ratings of the students on the Dreyfus scale were as follows: (1) they were new learners; (2) they were not yet competent; (3) they demonstrated competence; (4) they were proficient; and (5) they were functioning above the level of proficiency (i.e., as experts).

Unlike the Likert scale-based feedback among the regular cohort, the Dreyfus scale, with its concepts of the continuum of development along the path of expertise, was emphasized among the intervention cohort (Table 3).

#### Baseline and end-of-course GOSCE

For the students in both the regular and intervention cohorts, the 3 stations on the baseline GOSCE were the basic, head-to-toe, and thoracic systems, whereas the 3 stations on the end-of course GOSCE focused on the gastrointestinal, neuromuscular, and musculoskeletal systems. Within a pre-set scenario, the GOSCE was designed as a formative experience where students were asked to show their interviewing and PE skills on SPs. In both the regular and intervention cohorts, 18 groups of 9 students completed 3 different GOSCE stations in 3 identical circuits. All students worked together to show their competencies in each GOSCE, while 2 instructors assessed group performance and gave feedback according to the items in Table 3.

#### Introduction of Dreyfus scale-based feedback in the intervention cohort

One month before the implementation of Dreyfus scale-based feedback, the meaning and differentiating points for each level on the Dreyfus scale were introduced to the instructors. Instructional sessions, interactive workshops, and videotaped scenario-based ratings were arranged for training in the performance dimension and frame of reference. In those 2-hour sessions, the necessary knowledge and defined behavioral examples associated with the different ratings on the Dreyfus scale were presented [6,7]. For the students, the meanings of each level on the Dreyfus scale, as a continuum of skills development, were presented by the instructors. Overall, similar training and rubrics were provided for instructors using either the Likert or Dreyfus scale.

#### Validity and reliability of the survey tool

Upon evaluation by 2 experts, the content validity index (CVI) of the 6 Likert scale-based items was from 0.8 to 0.94 (Table 2). The total scale-level CVI (S-CVI) was 0.87, indicating that the experts considered the scale to have excellent relevance for training objectives. Similarly, the item-level CVI of the 10 Dreyfus scale-based items ranged from 0.64 to 0.89 (Table 3). Item 1 was rated lower (0.64) than the others, indicating that it was less reliable than the others. The total S-CVI was 0.84, which suggests good reliability of the scale for training objectives. In general, both the Likert and Dreyfus scale-based assessments (Tables 2, 3) demonstrated good reliability (internal consistency), with Cronbach α coefficients of 0.79 and 0.82, respectively.

#### In-class evaluation of the class climate by SPs in the regular and intervention cohorts

Two senior SPs, who had more than a year of experience with our course, were placed in each small group. These SPs were responsible for observing the class climate in both the regular and intervention cohorts. The mean age (41.1±8.9 years versus 37.9±10 years) of the SP observers was not significantly different between the regular and intervention cohorts. The SPs were informed that the purpose of the study was to evaluate the class climate created by the instructors, but remained uninformed regarding the study design. The SP observers were trained for acceptable inter-rater reliability. The Flanders system was used to analyze the interactions of the verbal behaviors of the small groups’ instructors and students [8]. In our study, 2 (teacher indirect and student initiation) of the 5 (teacher indirect, teacher-directed, student response, student initiation, and silence) domains of the Flanders system were selected to evaluate class climate.

The SPs assessed their agreement on whether a good class climate was created by instructors through evaluating students in terms of 2 parameters (with a 5-point scale for each, yielding a range of 2–10) in the in-class observations (teacher indirect and student initiation). The teacher indirect parameter refers to teacher behaviors intended to stimulate and encourage student input, or praise and encouragement of students, whereas the student initiation parameter refers to students responding well to the directions or questions of their teacher by introducing their own ideas.

#### Self-evaluation of medical students in the regular and intervention cohorts

The Preparation for Hospital Practice Questionnaire (PHPQ) is a valid and reliable self-reporting questionnaire that contains 8 subscales (interpersonal skills, readiness/coping, collaboration, patient management and practical skills, understanding science, prevention, holistic care, and SDL). It is designed to assess key areas of medical hospital practice in different clinical settings [9]. From the sub-scales of the PHPQ, in this study, the items assessing SDL capabilities (evaluation of performance, identification of learning needs) were selected and completed by all students (n=162) at the end of the course (Table 4).

### Data analysis

The 2-sample Student t-test was used to compare various parameters between the regular and intervention cohorts.

## Results

### Baseline characteristics of instructors

Instructors’ mean age, gender distribution, distribution of academic degrees, affiliation, and prior experience of participation were not significantly different between the regular and intervention cohorts (Table 1).

### Dreyfus scale-based feedback enhanced students’ satisfaction with the instructors and the course, and created a better class climate

**Fig. 1.**
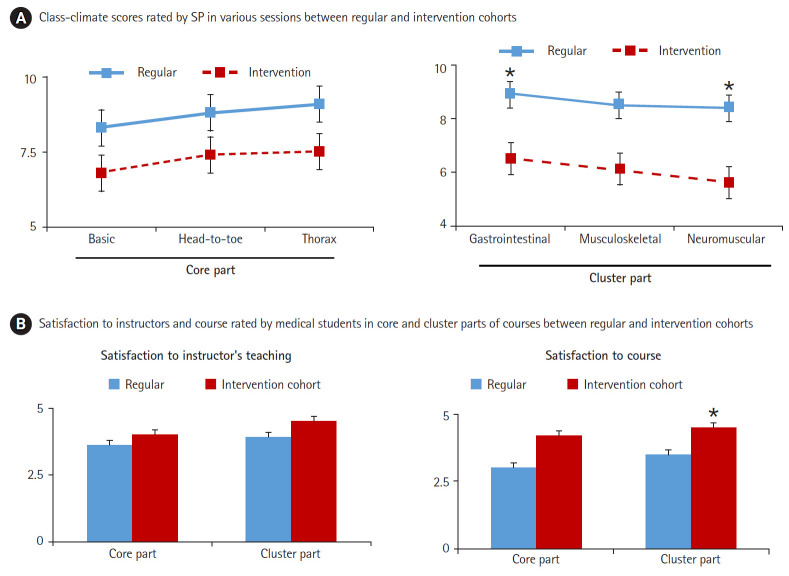
(A) SPs’ ratings of the class climate in the regular and intervention cohorts. (B) Students’ satisfaction with the instructors and course in the core and cluster parts of the course in the regular and intervention cohorts (18 groups per cohort, from either the 2015–2016 or 2017–2018 classes). SP, standardized patient. *P<0.01 versus data of the regular cohort.

The class-climate scores were not significantly different between the regular and intervention cohorts for the core part (basic, head-to-toe, thorax) of the interviewing and PE skills course (Fig. 1). In the regular cohort, as shown in Fig. 1A, the class climate scores were lower in the cluster part (gastrointestinal, musculoskeletal, and neuromuscular systems). In the intervention cohort, the implementation of Dreyfus scale-based feedback helped instructors to maintain a good class climate even in the more difficult cluster part of the sequential interview and PE sessions (Table 5). A higher class climate score for the cluster part was noted among the intervention cohort than among the regular cohort.

In the regular cohort, students’ satisfaction with the instructors and course was lower in the cluster part than in the core part (Fig. 1). In contrast, student satisfaction with the instructors and course was equal between the core and cluster parts in the intervention cohort. Parallel to the intervention’s positive effects on the class climate, better student satisfaction with the teaching quality of instructors and the course was noted in the intervention cohort compared with the regular cohort.

### Dreyfus scale-based feedback improved students’ performance on the end-of-course GOSCE in the intervention cohort

**Fig. 2.**
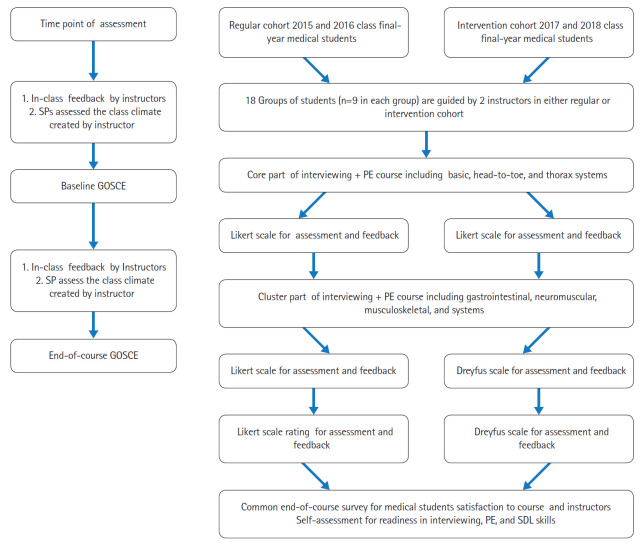
Schematic diagram of the study. SP, standardized patient; PE, physical examination; GOSCE, group objective structured clinical examination; SDL, self-directed learning.

The complexity of the skills developed in the cluster part of the interview and PE sessions was higher than that of the skills developed in the core part. As shown in Fig. 2, the baseline GOSCE was administered after the core part, whereas the end-of-course GOSCE was held after the cluster part. The performance of students in the regular cohort on the baseline GOSCE was better than their performance on the end-of-course GOSCE. There was no significant difference between the regular and intervention cohorts on the baseline GOSCE (regular: 84.5±3.1 versus intervention: 85.1±2.7). However, the intervention cohort showed better performance than the regular cohort on the end-of-course GOSCE (regular: 72.5±4.2 versus intervention: 86.4±3.9, P<0.01).

### Dreyfus scale-based feedback increased students’ readiness for interviewing, PE, and SDL skills in the intervention cohort

At the end of the sessions, students were asked to assess their readiness for interviewing, PE, and SDL skills. For PE skills, students’ readiness was higher in the intervention cohort than in the regular cohort (regular: 5.4±1.5 versus intervention: 8.7±1.8, P<0.01). Similarly, for SDL skills, the intervention cohort had higher readiness than the regular cohort (regular: 15.8±2.4 versus intervention: 26.1±2, P<0.001).

## Discussion

### Key results

The Dreyfus model has been applied in medicine, nursing, and public health to provide landmarks for professional development [6,7]. The present study examined whether Dreyfus scale-based feedback provided by trained instructors on interviewing and PE skills was more effective than Likert scale-based feedback. To achieve this aim, the Dreyfus scale was implemented in the intervention cohort for the complex cluster part of the interviewing and PE skills course. The effectiveness of this new intervention was assessed by the class climate (SPs), end-of-course GOSCE (instructors), satisfaction with the instructors and course (students), and self-assessment of readiness in skills and SDL (students), and the results were successful.

### Comparison with previous relevant studies

Multimedia-assisted simulations can improve the effectiveness of PE training [10]. Notably, video and SP simulations were used in our course to enhance the effectiveness of PE training. In the objective structured clinical observation (OSCE) of final-year medical students and postgraduate year 1 residents, as for the results of the United States Medical Licensing Examination (USMLE) step 2, the PE scores were significantly lower (59.6%) than the scores for history-taking [11,12]. Therefore, in this study, we provided repeated hands-on PE practice for each medical student in different sessions of the core and cluster parts of the course. Additionally, in both the OSCE and USMLE step 2, the mean scores of examinees in the cluster part, including PE skills of the neuromuscular and musculoskeletal systems, were lower than those in the core part, including cardiovascular, respiratory, and gastrointestinal systems [11,12]. Therefore, to smooth out the PE training from simple to complex, the sessions in our study were arranged in order of core to cluster, including the basic, head-to-toe, thoracic, gastrointestinal, neuromuscular, and musculoskeletal systems [12]. Notably, the appropriateness of the conceptual framework of our year-long clinical interviewing and PE course was supported by positive feedback from instructors and learners in both the regular and intervention cohorts.

Appropriate evaluations, feedback, and scoring can create a good class climate by facilitating interactions between clinical instructors and medical learners [13]. For healthcare providers, interviewing and PE skills training and learning must be ongoing. Specific evaluation and feedback by instructors support students’ engagement and self-reflection. For small-group sessions in clinical interviewing and PE skills courses, a structured format is necessary for the establishment of consensus among instructors. Likert scale-based feedback and scoring did not provide students with knowledge about their skill development levels, whereas Dreyfus scale-based feedback and scoring clearly provided students with this knowledge [14]. In this study, by promoting more interaction in the intervention cohort, Dreyfus scale-based feedback helped instructors to create a better class climate compared to the regular cohort, in which Likert scale-based feedback was given.

### Interpretation and suggestions

In this study, using a scale ranging from 1=poor to 5=excellent, the regular cohort participants reported that Likert scale-based feedback did not satisfy their desire to know how much their clinical skills had advanced. Instead, using a scale of 1=novice, 2=advanced beginner, 3=competent, 4=proficient, and 5=expert, the intervention cohort participants reported that Dreyfus scale-based feedback helped them understand how their skills had progressed. Additionally, the Likert scale provided feedback on only 6 areas, whereas the Dreyfus scale used in our study provided feedback on 10 areas. That may also account for increased satisfaction, because the students received more detailed feedback with the Dreyfus scale. The use of a rubric with the Dreyfus scale may have allowed more discussion points than was possible using the Likert scale.

Both real patients and SPs are involved in the interviewing and PE skills training of medical students. In our small group-based clinical skills course, SPs were trained to present specific complaints. Furthermore, inter-rater reliability in the SP assessment was good across a 4-year observation period, with kappa values of 0.61, 0.69, 0.72, and 0.7. This was a pilot study of the involvement of SPs in the evaluation of the class climate in a small group-based interviewing and PE course, and their inclusion showed good results.

SDL enables health professionals to update their knowledge continuously during their careers [3]. It has been reported that assessments such as OSCE stimulate students to shift from just-in-time learning to a longitudinal pattern of SDL. Evaluations and feedback are powerful driving forces for student’s SDL. Professional landmark-based evaluations and feedback are key features for effective clinical teaching and learning. In line with a previous study, structuralized Dreyfus scale-based feedback enhanced medical students’ SDL skills in our study [15]. Additionally, an appropriate assessment and feedback format motivated instructors to maximize their teaching by creating a good class climate.

### Limitation

A limitation of this 4-year prospective interventional study is that in our new course, the positive effects of the new intervention were only observed in the performance of medical students with SPs. Our study did not assess the long-term effects of this new intervention on patient outcomes. Based on this initial encouraging result, it is mandatory to confirm the effectiveness of this intervention in a clinical setting with real patients in a future study.

### Conclusion

This interventional study confirmed the benefits of implementing Dreyfus scale-based feedback in the complex cluster part of our interviewing and PE course for final-year medical students. The benefits include the creation of a good class climate, an increase in student satisfaction with instructors and the course, and enhancement of student performance by simulating SDL and engagement. Furthermore, the effects of using the Dreyfus scale, which has elements of continuous professional development, instead of the Likert scale for feedback in the cluster part of training were plausible.

## Figures and Tables

**Table 1.** Baseline characteristics of instructors in the regular and intervention cohorts

Characteristic	Regular cohort: 2015 and 2016 classes (n=36)	Intervention cohort: 2017 and 2018 classes (n=36)
Age (yr)	43.8±5.9	46.3±8.6
Male	67	64
Lecturer	36	28
Assistant professor	39	39
Associate professor	22	28
Professor	3	5
Affiliation of instructors		
Community-based	31	33
Hospital-based	69	66
Prior participation in workshop on teaching and evaluation of interviewing and physical examination skills	80	78

Values are presented as mean±standard deviation or %.

**Table 2.** Content of the Likert scale that instructors used for in-class, baseline GOSCE, and end-of-course

Variable	Items	Range of score
Goals of interviewing	Doing a comprehensive interview	3–15
	Performing an interview with a case-based focus	
	Overall time management for interviewing	
Goals of the PE	Performing a full PE	3–15
	Performing a focused PE	
	Overall time management for PE	

GOSCE evaluation and feedback for practicing skills in interviewing and PE in the regular cohort. Rating on the Likert scale: 1=poor to 5=excellent for students’ performance. The scores were converted to percentages for comparison of the baseline GOSCE including interviewing and PE skills of the basic, head-to-toe, and thoracic systems with the end-of-course GOSCE focused on interviewing and PE skills of the gastrointestinal, musculoskeletal, and neuromuscular systems. GOSCE, group objective structured clinical examination; PE, physical examination.

**Table 3.** Content of the Dreyfus scale that instructors used for in-class, baseline GOSCE, and end-of-course

Variable	Items	Range of score
Doing a comprehensive clinical interview	1. Communication/care skills	5–25
	2. Interview techniques	
	3. Appropriateness of interviewing sequence	
	4. Time management for interviewing	
	5. Symptom- and laboratory data-based interviewing	
Performing a full PE	6. Examination/care skills	5–25
	7. PE techniques	
	8. Appropriateness of PE sequence	
	9. Time management for PE	
	10. Symptoms and laboratory data-based PE	

GOSCE evaluation and feedback for practicing skills in interviewing and PE in the intervention cohort. Performance on the Dreyfus scale: 1=new learners, 2=not yet competent, 3=competence, 4=proficiency, and 5=expert-level performance. The score was converted to a percentage for comparison. GOSCE, group objective structured clinical examination; PE, physical examination.

**Table 4.** End-of-course self-evaluation of readiness for interviewing, PE, and SDL for both the regular and intervention cohorts

Subscale	Items (I am…)	Range of score
SDL readiness subscale of Preparation for Hospital Practice Questionnaire	1. Well-prepared for taking responsibility for my own learning	6–36
	2. Well-prepared for continually evaluating my own performance	
	3. Well-prepared for evaluating my educational experience	
	4. Well-prepared for investing time in developing my skills	
	5. Well-prepared for identifying my own educational needs	
	6. Well-prepared for keeping up to date with medicine	
Readiness for interviewing and PE	1. Performing a comprehensive clinical interviewing	2–10
	2. Performing a full PE	

Rating on the Likert scale: 1=poor to 5=excellent. The score was converted to a percentage for comparison. PE, physical examination; SDL, self-directed learning.

**Table 5.** The end-of-course assessment of class climate by SPs and students’ satisfaction

	SP-assessed mean score of instructor-created class climate	Average score for course satisfaction for the core and cluster parts	Average scores for the core and cluster parts for the 5 aspects of satisfaction with instructors’ teaching quality
Regular cohort	6.5±1.2	3.24±0.37	3.82±0.57
Intervention cohort	8.7±1.05^*^	4.61±1.12^**^	4.48±1.13^*^

Values are presented as mean±standard deviation. Likert scale, 1 to 5: 1=poor to 5=excellent. SP, standardized patient. *P<0.05 or **P<0.01 vs. the regular cohort (18 groups in each cohort, from either the 2015–2016 or 2017–2018 classes).

## Supplementary materials

Supplementary materials are available from Harvard Dataverse: https://doi.org/10.7910/DVN/47WSRS

Supplement 1. Audio recording of the abstract.
